# Incidence and predictors of tuberculosis among HIV-positive adults on antiretroviral therapy at Debre Markos referral hospital, Northwest Ethiopia: a retrospective record review

**DOI:** 10.1186/s12889-019-7912-9

**Published:** 2019-11-27

**Authors:** Belisty Temesgen, Getiye Dejenu Kibret, Nakachew Mekonnen Alamirew, Mamaru Wubale Melkamu, Yitbarek Tenaw Hibstie, Pammla Petrucka, Animut Alebel

**Affiliations:** 1 Debre Markos Referral Hospital, Debre Markos, Ethiopia; 2 College of Health Science, Debre Markos University, P.O. Box 269, Debre Markos, Ethiopia; 3 College of Nursing, University of Saskatchewan, Saskatoon, Canada; 4 School of Life Sciences and Bioengineering, Nelson Mandela African Institute of Science and Technology, Arusha, Tanzania; 5 Faculty of Health, University of Technology Sydney, Sydney, Australia

**Keywords:** Debre Markos, HIV, Incidence, Predictors, Tuberculosis

## Abstract

**Background:**

Tuberculosis is the leading cause of morbidity and mortality among people living with human immunodeficiency virus. Almost one-third of deaths among people living with human immunodeficiency virus are attributed to tuberculosis. Despite this evidence, in Ethiopia, there is a scarcity of information regarding the incidence and predictors of tuberculosis among people living with HIV. Thus, this study assessed the incidence and predictors of tuberculosis among HIV-positive adults on antiretroviral therapy.

**Methods:**

This study was a retrospective record review including 544 HIV-positive adults on antiretroviral therapy at Debre Markos Referral Hospital between January 1, 2012 and December 31, 2017. The study participants were selected using a simple random sampling technique. The data extraction format was adapted from antiretroviral intake and follow-up forms. Cox-proportional hazards regression model was fitted and Cox-Snell residual test was used to assess the goodness of fit. Tuberculosis free survival time was estimated using the Kaplan-Meier survival curve. Both the bi-variable and multivariable Cox-proportional hazard regression models were used to identify predictors of tuberculosis.

**Results:**

In the final analysis, a total of 492 HIV-positive adults were included, of whom, 83 (16.9%) developed tuberculosis at the time of follow-up. This study found that the incidence of tuberculosis was 6.5 (95% CI: 5.2, 8.0) per 100-person-years (PY) of observation. Advanced World Health Organization clinical disease stage (III and IV) (AHR: 2.1, 95% CI: 1.2, 3.2), being ambulatory and bedridden (AHR: 1.8, 95% CI: 1.1, 3.1), baseline opportunistic infections (AHR: 2.8, 95% CI: 1.7, 4.4), low hemoglobin level (AHR: 3.5, 95% CI: 2.1, 5.8), and not taking Isonized Preventive Therapy (AHR: 3.9, 95% CI: 1.9, 7.6) were found to be the predictors of tuberculosis.

**Conclusion:**

The study found that there was a high rate of tuberculosis occurrence as compared to previous studies. Baseline opportunistic infections, being ambulatory and bedridden, advanced disease stage, low hemoglobin level, and not taking Isonized Preventive Therapy were found to be the predictors of tuberculosis. Therefore, early detection and treatment of opportunistic infections like tuberculosis should get a special attention.

## Background

Tuberculosis (TB) is the leading cause of morbidity and mortality among People Living with Human Immunodeficiency Virus (PLHIV) [1]. PLHIV are 20 times more likely to develop TB as compared to those without HIV [2, 3]. In 2017, an estimated 10 million people developed TB with 1.6 million succumbing to the disease, of whom, 9% were PLHIV. In the same year, approximately 300,000 people died due to Acquired Immunodeficiency Syndrome (AIDS)-related TB worldwide, with 72% of these deaths occurring in Africa [4]. Sub-Saharan Africa (SSA) bears the highest (74%) burden of all TB cases [5, 6].

Ethiopia is one of the SSA countries with the highest prevalence of TB/HIV co-infection and ranked seventh among the world’s 30 highest TB burden countries. In 2017, according to the Centers for Diseases Control (CDC) and Prevention report, the incidence rate of TB in Ethiopia was 164 cases per 100,000 population including approximately 7% who were PLHIV. In the same year, the mortality rate of TB patients in Ethiopia was 24 per 100,000 population [7]. A range of interventions have been implemented at the global and local levels to tackle this challenge. For example, integrated management of TB/HIV co-infection in a single health facility via using a single health care provider to deliver integrated therapy and manage both diseases efficiently. In addition, a scale-up of Antiretroviral Therapy (ART) was introduced aimed to reduce HIV-related morbidity and mortality as well as to increase the survival of HIV-infected patients [8–11].

The Ethiopian government has adapted and implemented different strategies to reduce TB related morbidity and mortality among PLHIV. For example, the Ministry of Health (MOH) decentralized TB/HIV co-infection care services from hospitals to health centers and strengthened referral systems to improve TB care and treatment at the community level [12]. In addition, early initiation of ART and early screening of TB, prior to provision of Isonized Preventive Therapy (IPT) [13] and Co-trimoxazole Prophylactic Therapy (CPT), were interventions implemented to reduce the incidence of TB among PLHIV [14–18].

Although the incidence of TB among HIV-positive adults in Ethiopia has improved due to different interventions, it remains the major cause of death among PLHIV. Previous Ethiopian studies have documented that the incidence of TB among HIV-positive adults ranged from 3.3 per 100 PY of observation in Addis Ababa [19] to 8.6 per 100 PY of observation in Afar Region [20]. Previous Ethiopian-based studies have identified a number of contributing factors to increasing TB occurrence among HIV-positive adults including household family size between three to four individuals, cigarette smoking, not taking IPT, not taking CPT, low baseline CD4 counts, advanced World Health Organization (WHO) clinical disease stages (Stage II or IV), and having a history of diabetic mellitus [20–22].

The Ethiopian government targeted a reduction of TB related deaths by 90% and TB incidence by 80% by 2030 from 2015 levels [23]. To evaluate this ambitious plan, current and up-to-date information related to the occurrence of TB is vital. Therefore, this retrospective record review explored the incidence of TB among HIV-positive adults at Debre Markos Referral Hospital. Results obtained from this study will contribute to evidence for policy makers and program planners working at various levels of TB control programs, as well as informing health care professionals in the areas of TB control and prevention.

## Methods

### Study design, area, and period

A retrospective record review was conducted at Debre Markos Referral Hospital of applicable patient records from between January 1, 2012 and December 31, 2017. Debre Markos Referral Hospital is located in Debre Markos Town, which is located 299 km from Addis Ababa, the capital city of Ethiopia and 265 km from Bahir-Dar, the main city of Amhara Regional State. The hospital serves more than 3.5 million people in East Gojjam Zone and neighboring areas. Apart from other services, the hospital has been providing ART follow-up care services since 2005. In the hospital, the recorded number of HIV-positive people ever started ART was 3716, of whom, 1, 569 HIV-positive adults started ART care between January 1, 2012 and December 31, 2017.

### Population

All HIV-positive adults ever started ART at Debre Markos Referral Hospital and who had at least 1 month of ART follow-up from January 1, 2012 to December 31, 2017 were the target population for this study. All HIV-positive adults ever started ART from January 1, 2012 to December 31, 2017, however, HIV-positive adults on ART who had TB at the beginning of the follow-up and those who had incomplete baseline data for important variables (i.e., WHO stage, CD4 counts, hemoglobin, (Hgb), IPT, CPT and level of ART adherence) were excluded from the study.

### Sample size determination and sampling procedures

The minimum required sample size for this study was calculated using a sample size determination formula for survival analysis using Stata™ Version 13 statistical software by considering CD4 count, functional status, and WHO clinical staging as major exposure variables. It was calculated by considering the following statistical assumptions: two-sided significant level (α) of 5%, power 80%, Z_a/2_ = value at 95% CI =1.96, q_1_: proportion of subjects that are in group 1 (exposed), q_0:_ proportion of subjects that are in group 2 (unexposed); 1-q_1_, HR: hazard ratio, and probability of event (E) for functional status were taken from a study conducted at the University of Gondar Teaching Hospital (0.33) [21]. Finally, the calculated sample size for this study was 544. To select the study participants, the records of all HIV-positive adults ever started ART and recorded from January 1, 2012 to December 31, 2017 were sorted. Then, the study participants were selected using a simple random sampling technique through computer-generated numbers. We selected this follow-up period for two reasons:: first, we intended to have a nearest 6 years of follow-up; and second, to take advantage of standardized ART documentation and reporting formats used during this period.

### Data collection tool and procedures

The data extraction tool was prepared from the ART entry and follow-up forms. To ensure data quality, before data collection, the data extraction tool was prepared carefully from a standardized ART intake and follow-up forms. Furthermore, before the beginning of data collection, we verified consistency between data recording systems and the prepared checklist by randomly selecting and completing a few chart reviews which resulted in minor amendments of the data collection tool. Three BSc nurses who have been working in the ART clinic of Debre Markos Referral Hospital collected the data. Two days of training was given for both data collectors and supervisor concerning the data collection tool and collection process. The supervisor and principal investigators performed a strict follow-up and supervision throughout the entire data collection period. The most recent laboratory test results before starting ART were considered as a baseline value. If there were no pre-treatment laboratory test results, obtained at the time of ART initiation, test results done within 1 month of ART initiation were used as a baseline data. In case of two results obtained within a month, the mean value was computed and taken as a baseline.

### Measurements

The dependent variable was the time to develop TB. The predictor variables were: Socio-demographic characteristics (age, sex, marital status, residence, family size, level of education, and occupation), Baseline clinical and laboratory characteristics (WHO clinical stage, CD4 cell count, hemoglobin level, history of TB, and history of opportunistic infections (OIs) and body mass index (BMI), and ART and other medication-related characteristics (type ART regimens, regimen change, ART side effects, ART adherence, IPT, and CPT).

The focus of this study was on health care documentation of the event (the occurrence of TB) for HIV-positive adults in cases where TB developed after ART initiation until the end of the study. This was ascertained by review of patient records. However, the study participants were classified as censored in either of the following conditions: if lost to follow-up or died before developing TB or if alive at the end of the study, but didn’t develop TB and took anti-TB medications. These elements were ascertained by reviewing patient records. In this study, lost to follow-up was defined as an HIV-positive patient missing an ART appointment for one to 3 months [24]. Additionally, adherence was classified as good, fair, or poor, according to the percentage of drug dosage calculated from a monthly total dose of ART drugs; hence, good was reported if equal to or greater than 95% or ≤ 3 dose missing per month, fair if 85–94% or 4–8 dose missing per month, or poor if less than 85% or ≥ 9 dose missing per month [25]. Furthermore, low hemoglobin level was defined as Hgb level less than to 10 g/dl. Finally, opportunistic infections were diagnosed if HIV-positive adults developed any morbidities after starting ART, as documented by the health care professionals.

### Data processing and analysis

Data were entered using EPI-data™ Version 4.2, and analyzed using STATA Version 13 statistical software. Patient’s follow-up characteristics for continuous data were described in terms of central tendency, dispersion, and frequency distribution for categorical data. At the end of follow-up, the outcome of each study participant was dichotomized into censored or event. The necessary assumption of Cox proportional hazard regression model was checked using Schoenfeld residual and Log-Log plot tests. In addition, the model goodness of fit was assessed using Cox-Snell residual test and model with the least value of Akaike’s information criteria selected as the best model. The Kaplan Meier survival curve was used to estimate the TB free survival time of HIV-positive adults on ART. Log rank test was used to compare the survival curves of different categorical explanatory variables. Bi-variable Cox-proportional hazard regression model was used to screen variables for the final model. Variables having *p*-value ≤0.25 in the bi-variable analysis were fitted into the multivariable Cox-proportional hazard regression model. Finally, adjusted hazard ratio with its corresponding 95% confidence interval was reported to declare the presence of significant association between the explanatory and outcome variables.

## Results

### Socio-demographic characteristics of participants

After removal of fifty-two (52) incomplete records, 492 HIV-positive adult charts were included in the final analysis. The median age of the entire cohort was 33.0 years (IQR: 27, 40 years). More than half (53.6%) of the study participants were female and more than three fourth (78.86%) of the participants disclosed their HIV status (Table 1).

**Table 1:** Socio-demographic characteristics of patients on chronic HIV care at Debre-Markos Referral Hospital, Northwest Ethiopia

Characteristics	Frequency (N)	Percentage (%)
Age		
15–24 year	62	12.6
25–34 year	199	40.4
35–44 year	151	30.7
> 45 year	80	16.3
Sex		
Male	228	46.3
Female	264	53.7
Marital status		
Single	66	13.4
Married	268	54.5
Divorced	126	25.6
Widowed	32	6.5
Religion		
Orthodox	477	97.0
Others	15	3.0
Educational status		
No formal education	145	29.5
Primary	115	23.4
Secondary	144	29.3
Tertiary	86	17.4
Not recorded	2	0.4
Occupation		
Employed	110	22.6
Unemployed	376	77.4
Residence		
Within catchment area	464	94.3
Out of catchment area	28	5.7
Disclosure status		
Disclosed	388	78.9
Not disclosed	104	21.1
Family size		
<=3	389	82.7
> 3	85	17.3

### Baseline clinical, laboratory, ART, and other medication-related information

Clinically, more than half (61.8%) of the study participants were classified as WHO clinical stage I/II. The mean baseline CD4 cell count of the study participants was 252.7 cell /mm^3^ (SD: ± 9.5 cell /mm^3^). In addition, the majority (90.43%) of study participants had a Hgb of 10 g/dl and more. Almost, one-third (32.93%) of the participants had baseline OIs. Regarding functional status, the majority (81.2%) of study participants were classified as working functional status. At baseline, less than one-third (29.47%) of the participants were undernourished (BMI < 18.5). About 5% of the participants had a history of initial regimen change during follow-up. The majority (95.53%) of participants had a history of good adherence. Moreover, the majority (85.98%) of participants took CPT; however, only 36.38% received IPT (Table 2).

**Table 2:** Baseline clinical, Laboratory, ART and other medication related information of HIV patients on chronic HIV care at Debre-Markos Referral Hospital, Northwest Ethiopia

Characteristics	Frequency (N)	Percentage (%)
WHO clinical staging		
I / II	304	61.8
III /IV	188	38.2
CD4 count		
< 100	113	23.0
100–200	123	25.1
201–350	140	28.5
> = 351	115	23.4
Functional status		
Working	418	85.1
Ambulatory /bed redden	73	14.9
Hemoglobin level		
< 10 g/dl	44	9.6
> = 10 g/gl	416	90.4
BMI/MUAC		
Underweight	145	29.5
Not Underweight	347	70.5
Eligible criteria		
CD4 cell count	199	40.5
WHO stage	49	10.0
Both	116	23.6
Test & treat	89	18.2
Not recorded	38	7.7
Initial regimen		
1d = ZDV-3TC EFV	11	2.2
1c = ZDV-3TC- NVP	36	7.3
1e = TDF-3TC-EFV	423	86.2
1f = TDF-3TF-NVP	15	3.1
Other	6	1.2
Past TB history		
Yes	7	1.4
No	483	98.6

### Incidence of tuberculosis

**Fig. 1:**
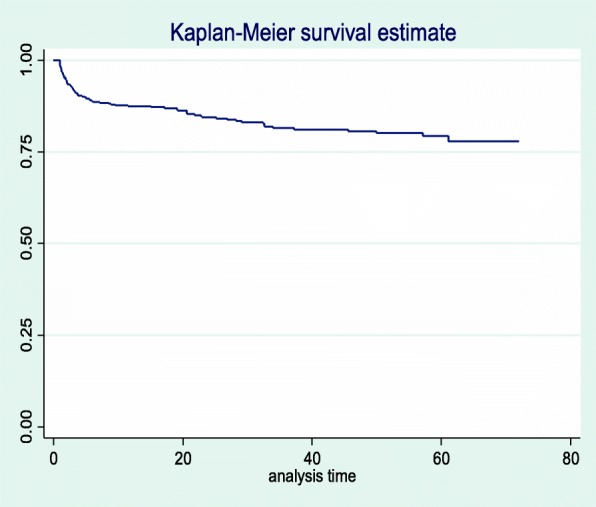
The overall Kaplan-Meier survival curve of TB free survival time of HIV-positive adults on ART car at Debre-Markos Referral Hospital, Northwest Ethiopia

In this study, a total of 492 study participants were followed for a different period, contributing a cohort of 1285.54 PY of observation. During the follow-up period, 83 (16.9%) of the study participants experienced TB with an overall incidence rate of TB of 6.5 (95% CI: 5.2, 8.0) per 100 PY of observation. A 14.5% TB incidence rate (95% CI: 11.3, 18.7 per 100 PY) was highest in the first year of follow-up, decreasing in subsequent years. The cumulative probability of TB free survival at the end of 1 year, two, three, four, five and 6 years were 0.87, 0.84, 0.82, 0.80, 0.79, and 0.78 respectively. The mean TB free survival time of the entire follow up was 60.8 months (95% CI: 58.2, 63.1 months) (Fig. 1).

### Bi-variable and multivariable cox-regression analysis

**Fig. 2:**
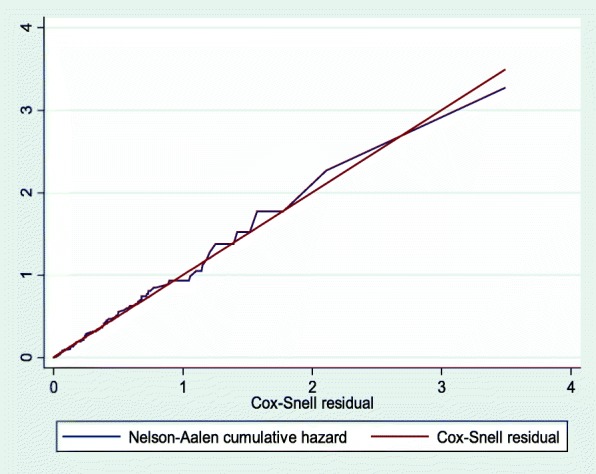
The goodness of fit test for Cox-proportional hazard regression model

Through multivariable Cox-regression analysis, advanced WHO clinical stages (III and IV), baseline OIs, being ambulatory and bedridden, low hemoglobin level (< 10 g/dl), and not taking IPT were found to be significant predictors of TB. In this study, the hazard of developing TB among HIV-positive adults on ART who were classified as WHO clinical stage III and IV at baseline was 2.1 (95% CI: 1.2, 3.2) times higher as compared to those classified as WHO Stages I and II. Similarly, the hazard of developing TB among HIV-positive adults classified as ambulatory and bedridden at baseline was 1.8 (95% CI: 1.1, 3.1) times higher as compared to those who were classified as working functional status. In addition, the hazard of developing TB among patients who had OIs at baseline was 2.8 times (95% CI: 1.7, 4.4) higher as compare to those who were OIs free at baseline. Furthermore, the hazard of developing TB among patients presenting with Hgb level less than 10 g/dl during ART initiation was 3.5 (95%CI: 2.1, 5.8) times higher than those with Hgb levels greater than or equal to 10 g/dl. Lastly, HIV-positive adults on ART who did not take IPT were 3.9 (95% CI: 1.9, 7.6) times more likely to develop TB as compared to those who took IPT (Table 3). The goodness of fit for the model was assessed using a Cox-Snell residual test (Fig. 2).

**Table 3:** Bi-variable and multivariable Cox-regression analysis to identify the predictors of tuberculosis among HIV positive adults on ART care at Debre-Markos Referral Hospital, Northwest Ethiopia

Variables	Survival status		CHR (95%CI)	AHR (95%CI)
	Event	Censored		
Sex				
Male	47	181	1.6(1.1 2.5)	1.2 (0.8, 2.0)
Female	36	228	1	1
CD4 cell count				
< 200 cell/mm^3^	55	180	2.2 (1.4, 3.4)	1.3 (0.8, 2.1)
> =200 cell/mm^3^	28	228	1	1
WHO clinical staging				
Stage I and II	26	278	1	1
Stage III and IV	57	131	4.0 (2.5, 6.4)	**2.1 (1.2, 3.2)**^**a**^
Functional status				
Working	51	367	1	**1**
Ambulatory & bedridden	32	41	5.0 (3.2, 7.8)	**1.8 (1.1, 3.1)**^**a**^
BMI / MUAC				
Underweight	32	113	1.6(1.1.2.6)	1.3 (0.8, 2.1)
Not Underweight	51	296	1	1
Hemoglobin level				
< 10 g/dl	27	20	6.7 (4.2, 10.7)	**3.5 (2.1, 5.8)**^**a**^
≥ 10 g/dl	56	383	1	1
Opportunistic infection				
Yes	51	111	3.7 (2.4,5.7)	**2.8 (1.7, 4.4)**^**a**^
No	32	298	1	**1**
CPT				
Yes	69	354	1	1
No	14	55	1.6 (0.9, 2.8)	1.4 (0.8, 2.5)
IPT				
Yes	10	169	1	1
No	73	240	4.8 (2.5, 9.3)	**3.9 (1.9, 7.6)**^**a**^

^a^Significant predictors and data are in bold

## Discussion

Tuberculosis is a major public health challenge and remains the most commonly diagnosed OI among PLHIV [3]. Therefore, we conducted this retrospective record review to determine the incidence and predictors of TB among HIV-positive adults at Debre Markos Referral Hospital. In this study, the overall incidence of TB among HIV-positive adults at Debre Markos Referral Hospital was 6.5 (95% CI: 5.2, 8.0) per 100 PY of observation. This finding is in line with previous Ethiopian studies conducted in Gondar (7.89 cases per 100 PY) [21] and Arba Minch (5.36 cases per 100 PY) [22]. On the other hand, this finding is higher than studies from developed as well as developing countries including: Brazil (2.28 cases per 100 PY) [26], Nigeria (0.57 cases per 100 PY) [27] and Tanzania (4.4 cases per 100 PY) [28].

The higher incidence of TB in this study may be attributed to the differences in follow-up period, sample size, characteristic of study participants, and difference in TB prevalence [18]. For example, the median follow-up period for a study conducted in Tanzania [28] was 2 years, whereas, in this study, it was 6 years. It is well understood that as the follow-up period increases, the number of events also increases. Additional possible explanation for the above variations could be due to the differences in characteristic of study participants. In this regard, a study done in Tanzania included all HIV-positive patients-initiated ART in all health facilities [28]. However, our study was conducted at a referral hospital providing tertiary level services for patients referred from health centers or general hospitals who are often experiencing advanced disease stage and are at higher risk of developing TB [29].

However, the incidence rate of TB obtained from this study is lower than a study conducted in Afar region, Northeast Ethiopia (8.6 cases per 100 PY) [20]. This variation could be explained by the difference in study settings, since this study was conducted at one referral hospital, whereas the study conducted in Afar region was done in three hospitals and two health centers. As the Ethiopian TB care strategy currently is decentralized patients commonly visit health centers before referred to hospitals; hence, including health centers as a study stetting will increase the case detection rate, which ultimately increases the incidence of TB [20]. Another possible explanation for this variation could be due to the difference in characteristics of study participants during ART initiation. In this study, about 38.2% of the study participants were classified as WHO clinical stage III or IV, but, in a study conducted in Afar health facilities, more than half (53.4%) of the study participants were classified as WHO stage III or IV [16]. As the WHO clinical disease stage becomes more advanced, the risk of OIs developing and recurring including TB simultaneously increases.

Regarding the time to develop TB, about 60% of TB cases developed TB in the first 6 months of follow-up. This finding is in line with studies conducted in different parts of Ethiopia and elsewhere [18, 20–22, 26–28]. The higher incidence of TB in early phase of ART could be associated with the progression of the disease from latent to active TB potentially remaining undetectable during early phase of ART [30]. Immune reconstitution inflammatory syndrome was very common in the first 2–12 weeks of ART. It highly increases the protective responses of the immune system, which leads to a typical inflammatory condition which creates a higher chance of latent TB infection to become active TB [31]. Moreover, the main actions of HAART are increasing CD4 cell counts and decreasing viral load, which ultimately improves the immune function and reduces the OIs occurrence including TB [32].

In this study, advanced WHO clinical disease stage (III and IV) was found to be an important predictor of TB among HIV-positive adults on ART. This finding is consistent with studies previously conducted in different regions of Ethiopia [21, 28, 29, 33–35], and South Africa [30]. as WHO clinical disease staging becomes more advanced, the risk of developing and recurrence of OIs including TB simultaneously increased [29].

Being ambulatory and bedridden was also another predictor of tuberculosis among HIV positive adults. Accordingly, those who were classified as ambulatory and bedridden were more likely to develop TB as compared to those who were classified as working. This finding is consistent with different studies conducted in Ethiopia [20, 21, 34]. Being bedridden and ambulatory functional status are more prone to develop TB because patients become bedridden and ambulatory due to advanced disease stage and more immune compromised stage of HIV [21]. The other possible explanation could be due to restriction from physical activities and inability to perform daily tasks which, indirectly, compromise the immune system [20] and may increase the risk of other OIs and TB.

In this study, patients who had baseline OIs were more likely to develop TB as compared with those without baseline OIs. This may be due to the presence of OIs during ART initiation will increases piles burden, which resulted from drug-drug interaction and side effects which, resulted synergic effects with HIV. This scenario contributes to further weakening of the immune system [30].

Moreover, patients who had a low baseline Hgb levels (< 10 g/dl) were more likely to develop TB as compared to those with normal Hgb levels (≥10 g/dl). Supportive findings were reported from previous studies conducted in different regions of Ethiopia [20–22, 34]. that the results in these studies found that advancing WHO disease stage presence of anemia also increased as a result of immunosuppression and reduction of bone marrow cell production [34].

Lastly, taking IPT was another independent predictor of tuberculosis among HIV positive adults on ART. Patients who had not taken IPT were more likely to develop TB as compared to those who took IPT. This finding is congruent with previous studies conducted in different parts of Ethiopia [15, 16, 20, 34], which may reflect the role of IPT in reducing the occurrence of TB among PLHIV [14, 36]. As a result, all HIV positive individuals who have no evidence of active TB infection should be taking IPT to prevent TB infection.

### Public health implications

The results of this study are a critical knowledge input for policy makers and program planners designing various TB control programs. Besides, results obtained from this study will be helpful for health care professionals working in the area of TB/HIV control and prevention unit of Debre Markos Referral Hospital. Additionally, the study provides insights into further intervention studies.

### Limitations of the study

This study has some limitations that must be considered before interpreting results. As the study was conducted through reviewing of records, it did not include important predictors of TB like housing conditions, household incomes, viral loads, and substance use due to incomplete recording system. In addition, study subjects who had incomplete data were excluded from the study. This could undermine or overestimate the incidence of TB. Furthermore, since this study was a facility-based study it does not capture HIV-positive individuals who are out of care (at the community level). The study also focused on adults only; hence, results might not be mirrored in pediatric patient groups.

## Conclusion

The study found a high rate of TB occurrence as compared to previous studies. Baseline OIs, being ambulatory and bedridden, advanced disease stage, low Hgb level, and not taking IPT were predictive of TB. Besides, a high rate of TB occurrence was observed in the first 6 months of ART follow-up. Therefore, based on our findings, we Suggest emphasis and close follow-up should be given for the first 6 months of ART follow-up. Moreover, early detection and treatment of OIs like TB should receive special attention. Furthermore, provision of IPT for HIV positive patients should be strengthened.

## Data Availability

The data sets used and/or analyzed during the current study are available from the Corresponding author on reasonable request.

## Abbreviations

AIDS: Acquired Immune Deficiency Syndrome
ART: Antiretroviral Therapy
CDC: Centers for Diseases Control and Prevention
CPT: Co-trimoxazole Preventive Therapy
HAART: Highly Active Antiretroviral Therapy
Hgb: Hemoglobin
HIV: Human Immunodeficiency Virus
PLHIV: People Living with Human Immunodeficiency Virus
TB: Tuberculosis
WHO: World Health Organization
